# Economic burden of cancer among patients with surgical resections of the lung, rectum, liver and uterus: results from a US hospital database claims analysis

**DOI:** 10.1186/s13561-017-0160-8

**Published:** 2017-06-02

**Authors:** Iftekhar Kalsekar, Chia-Wen Hsiao, Hang Cheng, Sashi Yadalam, Brian Po-Han Chen, Laura Goldstein, Andrew Yoo

**Affiliations:** 1 Medical Devices- Epidemiology, Johnson & Johnson, New Brunswick, NJ USA; 2 Franchise Health Economics and Market Access, Ethicon, Inc, Cincinnati, OH USA

**Keywords:** Surgical organ resections, Healthcare costs, Economic burden, Cancer surgery

## Abstract

**Objectives:**

To determine hospital resource utilization, associated costs and the risk of complications during hospitalization for four types of surgical resections and to estimate the incremental burden among patients with cancer compared to those without cancer.

**Methods:**

Patients (≥18 years old) were identified from the Premier Research Database of US hospitals if they had any of the following types of elective surgical resections between 1/2008 and 12/2014: lung lobectomy, lower anterior resection of the rectum (LAR), liver wedge resection, or total hysterectomy. Cancer status was determined based on ICD-9-CM diagnosis codes. Operating room time (ORT), length of stay (LOS), and total hospital costs, as well as frequency of bleeding and infections during hospitalization were evaluated. The impact of cancer status on outcomes (from a hospital perspective) was evaluated using multivariable generalized estimating equation models; analyses were conducted separately for each resection type.

**Results:**

Among the identified patients who underwent surgical resection, 23 858 (87.9% with cancer) underwent lung lobectomy, 13 522 (63.8% with cancer) underwent LAR, 2916 (30.0% with cancer) underwent liver wedge resection and 225 075 (11.3% with cancer) underwent total hysterectomy. After adjusting for patient, procedural, and hospital characteristics, mean ORT, LOS, and hospital cost were statistically higher by 3.2%, 8.2%, and 9.2%, respectively for patients with cancer vs. no cancer who underwent lung lobectomy; statistically higher by 6.9%, 9.4%, and 9.6%, respectively for patients with cancer vs. no cancer who underwent LAR; statistically higher by 4.9%, 14.8%, and 15.7%, respectively for patients with cancer vs. no cancer who underwent liver wedge resection; and statistically higher by 16.0%, 27.4%, and 31.3%, respectively for patients with cancer vs. no cancer who underwent total hysterectomy. Among patients who underwent each type of resection, risks for bleeding and infection were generally higher among patients with cancer as compared to those without cancer.

**Conclusions:**

In this analysis, we found that patients who underwent lung lobectomy, lower anterior resection of the rectum (LAR), liver wedge resection or total hysterectomy for a cancer indication have significantly increased hospital resource utilization compared to these same surgeries for benign indications.

## Background

Complex surgery and prolonged surgery time during organ resections can impair recovery and short-term outcomes, including extending hospital length of stay [1–3]. Additionally, surgical complexity and preoperative risk factors are associated with greater hospital costs [4, 5]. The National Surgical Quality Improvement Program of the American College of Surgeons has identified >50 preoperative risk factors, including comorbidities of cardiovascular disease, renal failure, cancer, and diabetes, and patient factors, such as age, body mass index, and prior chemo-radiation for risk stratification of patients undergoing surgery [6]. With differences in risk factors and perioperative situations, patients with cancer represent a distinct population of patients from those without cancer for surgical resection procedures [7]. Specifically among patients who underwent colon, rectal, and pancreatic resections for cancer, greater surgical complexity has been associated with worse outcomes in the 30 days following procedures [3]. Also, a study of 59,525 women who underwent hysterectomies reported a significantly longer operating time and a two-fold higher complication rate among women with gynecologic malignancies compared to women with benign conditions [8]. Better understanding of the current hospital and economic burden of technically challenging surgical resections may assist with assessing the value of new technologies that may reduce healthcare resource utilization and costs. The objectives of this study were to determine hospital resource utilization, the associated costs, and the frequency of complications during hospitalizations for four common surgical resections and to additionally estimate the incremental burden among patients with cancer undergoing these resections.

## Methods

### Study population

This study was cross-sectional in design and from a US hospital perspective. Patients were identified from the Premier Research Database between January 2008 and December 2014, who had any of the following types of elective surgical resections with open or minimally invasive approaches: lung lobectomy, lower anterior resection (LAR) of the rectum, wedge resection of the liver or total hysterectomy. The Premier Research Database contains complete clinical coding, hospital cost, and patient billing data from more than 600 hospitals throughout the US. Although the database excludes federally funded hospitals (e.g., Veterans Affairs), the hospitals included are nationally representative based on bed size, geographic region, location (urban/rural), and teaching status. The database contains a date-stamped log of all billed items by cost-accounting department including medications; laboratory, diagnostic, and therapeutic services; and primary and secondary diagnoses for each patient’s hospitalization. Additionally, the database also provides patient demographic and payer information. For all patients included in the four study groups, cancer status was determined based on the International Classification of Diseases, 9th revision, Clinical Modification (ICD-9-CM) diagnosis codes (lung cancer: 162.XX; colorectal cancer: 153.X, 154.X; liver cancer: 155.X; uterine, cervical, or adnexal cancer: 179.X, 180.X, 182.X, 183.X, and 184.X).

### Demographics, hospital, and clinical characteristics

Patient demographics and hospital characteristics evaluated during the index hospitalization for surgical resection included age, sex, marital status, race, payer type, surgical approach (if available), hospital geographic region, hospital teaching status, urban vs. rural hospital, hospital bed size and surgical volume, operating physician specialty, calendar year of surgery, and an indicator for whether hospital costs were derived from procedural hospital records or a cost to charge ratio. Patient comorbidities, identified by the presence of an ICD-9-CM code on hospital discharge records, were additionally evaluated and included diabetes, hypertension, obesity, alcohol abuse, cardiac arrhythmia, congestive heart failure, depression, hypothyroidism, AIDS/HIV, liver disease, pulmonary circulation disorders, peripheral vascular disease, rheumatoid arthritis, renal failure, valvular disease, cerebrovascular disease, and myocardial infarction.

### Outcome measurements

Hospital resource utilization in categories of operating room time (ORT), hospital length of stay (LOS), and total hospital costs (inflation adjusted to 2014 USD) were evaluated. Furthermore, the frequency of complications during hospitalizations was determined based on ICD-9-CM diagnosis codes and included general complications of bleeding and infection.

### Statistical analyses

Generalized estimating equation (GEE) models with the appropriate distribution and link functions tailored to the empirical distributions of each outcome were used to control for differences in patient, procedural and hospital characteristics in comparing outcomes between patients who underwent resections for cancer vs. non-cancer conditions. Adjusted outcomes were generated for each of the comparator groups using the least squares means approach. Covariates included in the GEE models were age, sex, marital status, race, payer type, procedural approach, patient comorbidities, urban vs. rural hospital, hospital teaching status, hospital geographic region, hospital bed size and procedure volume, operating physician specialty, calendar year of surgery, and hospital procedural costing type. Analyses were conducted among patients within the different resection groups accounting for the clustering of patients within hospitals. All analyses were conducted using SAS version 9.4. P-values of <0.05 (two-sided) were considered statistically significant.

Sensitivity analyses were conducted to test the robustness of the primary results. In lung lobectomy and total hysterectomy cohorts where data on surgical approaches were available, analyses were stratified by open versus minimally invasive approaches (i.e., lung lobectomy: video-assisted thoracoscopic surgery (VATS); hysterectomy: laparoscopic, laparoscopically assisted vaginal hysterectomy (LAVH), vaginal) to examine the incremental effect of cancer in specific approaches.

## Results

### Demographics, comorbidities, and hospital characteristics

Among the identified patients who underwent surgical resection, 23 858 (87.9% with cancer) underwent lung lobectomy, 13 522 (63.8% with cancer) underwent LAR, 2916 (30.0% with cancer) underwent liver wedge resection, and 225 075 (11.3% with cancer) underwent total hysterectomy. Patient demographics, comorbidities, and hospital characteristics are shown in Tables 1, 2 and 3, respectively, for the study groups with the different types of surgical resections and stratified by cancer status (yes vs. no).

**Table 1:** Demographics of the Study Groups with Surgical Resections

	Lung Lobectomy			LAR			Liver Wedge Resection			Total Hysterectomy		
	Overall *N* = 23 858 N (%)	Cancer *N* = 20 976 N (%)	No Cancer *N* = 2882 N (%)	Overall *N* = 13 522 N (%)	Cancer *N* = 8627 N (%)	No Cancer *N* = 4895 N (%)	Overall *N* = 2916 N (%)	Cancer *N* = 874 N (%)	No Cancer *N* = 2042 N (%)	Overall *N* = 225 075 N (%)	Cancer *N* = 25 511 N (%)	No Cancer *N* = 199 564 N (%)
Age Group (years)												
18–44	627 (2.6)	267 (1.3)	360 (12.5)	1132 (8.4)	553 (6.4)	579 (11.8)	441 (15.1)	59 (6.8)	382 (18.7)	92 256 (41.0)	2096 (8.2)	90 160 (45.2)
45–54	2447 (10.3)	1902 (9.1)	545 (18.9)	2838 (21.0)	1743 (20.2)	1095 (22.4)	548 (18.8)	131 (15.0)	417 (20.4)	75 514 (33.6)	4613 (18.1)	70 901 (35.5)
55–64	5859 (24.6)	5055 (24.1)	804 (27.9)	3591 (26.6)	2256 (26.2)	1335 (27.3)	808 (27.7)	273 (31.2)	535 (26.2)	29 410 (13.1)	8404 (32.9)	21 006 (10.5)
65–74	9104 (38.2)	8345 (39.8)	759 (26.3)	3487 (25.8)	2290 (26.5)	1197 (24.5)	749 (25.7)	268 (30.7)	481 (23.6)	19 222 (8.5)	6544 (25.7)	12 678 (6.4)
≥75	5821 (24.4)	5407 (25.8)	414 (14.4)	2474 (18.3)	1785 (20.7)	689 (14.1)	370 (12.7)	143 (16.4)	227 (11.1)	8673 (3.9)	3854 (15.1)	4819 (2.4)
Sex												
Female	12 427 (52.1)	10 818 (51.6)	1609 (55.8)	6770 (50.1)	3738 (43.3)	3032 (61.9)	1540 (52.8)	304 (34.8)	1236 (60.5)	225 075 (100.0)	25 511 (100.0)	199 564 (100.0)
Male	11 431 (47.9)	10 158 (48.4)	1273 (44.2)	6752 (49.9)	4889 (56.7)	1863 (38.1)	1376 (47.2)	570 (65.2)	806 (39.5)	0	0	0
Marital Status												
Married	12 716 (53.3)	11 098 (52.9)	1618 (56.1)	7363 (54.5)	4653 (53.9)	2710 (55.4)	1612 (55.3)	485 (55.5)	1127 (55.2)	126 972 (56.4)	11 958 (46.9)	115 014 (57.6)
Single	8745 (36.7)	7812 (37.2)	933 (32.4)	4399 (32.5)	2936 (34.0)	1463 (29.9)	969 (33.2)	265 (30.3)	704 (34.5)	78 478 (34.9)	10 667 (41.8)	67 811 (34.0)
Other	2397 (10.1)	2066 (9.9)	331 (11.5)	1760 (13.0)	1038 (12.0)	722 (14.8)	335 (11.5)	124 (14.2)	211 (10.3)	19 625 (8.7)	2886 (11.3)	16 739 (8.4)
Race												
African American	1689 (7.1)	1505 (7.2)	184 (6.4)	802 (5.9)	575 (6.7)	227 (4.6)	316 (10.8)	86 (9.8)	230 (11.3)	33 344 (14.8)	2307 (9.0)	31 037 (15.6)
White	18 457 (77.4)	16 287 (77.7)	2170 (75.3)	9987 (73.9)	6207 (72.0)	3780 (77.2)	1821 (62.5)	438 (50.1)	1383 (67.7)	145 413 (64.6)	17 874 (70.1)	127 539 (63.9)
Other	3712 (15.6)	3184 (15.2)	528 (18.3)	2733 (20.2)	1845 (21.4)	888 (18.1)	779 (26.7)	350 (40.1)	429 (21.0)	46 318 (20.6)	5330 (20.9)	40 988 (20.5)
Payer Type												
Commercial	6958 (29.2)	5624 (26.8)	1334 (46.3)	6467 (47.8)	3786 (43.9)	2681 (54.8)	1319 (45.2)	312 (35.7)	1007 (49.3)	149 825 (66.6)	11 834 (46.4)	137 991 (69.2)
Medicaid	1191 (5.0)	1018 (4.9)	173 (6.0)	623 (4.6)	448 (5.2)	175 (3.6)	324 (11.1)	142 (16.3)	182 (8.9)	23 763 (10.6)	1564 (6.1)	22 199 (11.1)
Medicare	14 767 (61.9)	13 568 (64.7)	1199 (41.6)	5735 (42.4)	3883 (45.0)	1852 (37.8)	1113 (38.2)	390 (44.6)	723 (35.4)	32 780 (14.6)	10 241 (40.1)	22 539 (11.3)
Other	942 (4.0)	766 (3.7)	176 (6.1)	697 (5.2)	510 (5.9)	187 (3.8)	160 (5.5)	30 (3.4)	130 (6.4)	18 707 (8.3)	1872 (7.3)	16 835 (8.4)

**Table 2:** Comorbidities of the Study Groups with Surgical Resections

	Lung Lobectomy			LAR			Liver Wedge Resection			Total Hysterectomy		
	Overall *N* = 23 858 N (%)	Cancer *N* = 20 976 N (%)	No Cancer *N* = 2882 N (%)	Overall *N* = 13 522 N (%)	Cancer *N* = 8627 N (%)	No Cancer *N* = 4895 N (%)	Overall *N* = 2916 N (%)	Cancer *N* = 874 N (%)	No Cancer *N* = 2042 N (%)	Overall *N* = 225 075 N (%)	Cancer *N* = 25 511 N (%)	No Cancer *N* = 199 564 N (%)
Comorbidities												
Diabetes	4805 (20.1)	4291 (20.5)	514 (17.8)	2394 (17.7)	1726 (20.0)	668 (13.7)	652 (22.4)	270 (30.9)	382 (18.7)	21 034 (9.4)	5560 (21.8)	15 474 (7.8)
Hypertension	14 719 (61.7)	13 233 (63.1)	1486 (51.6)	6974 (51.6)	4585 (53.2)	2389 (48.8)	1507 (51.7)	540 (61.8)	967 (47.4)	65 648 (29.2)	13 764 (54.0)	51 884 (26.0)
Obesity	2546 (10.7)	2152 (10.3)	394 (13.7)	1620 (12.0)	983 (11.4)	637 (13.0)	365 (12.5)	93 (10.6)	272 (13.3)	32 775 (14.6)	7353 (28.8)	25 422 (12.7)
Alcohol Abuse	745 (3.1)	695 (3.3)	50 (1.7)	262 (1.9)	187 (2.2)	75 (1.5)	80 (2.7)	54 (6.2)	26 (1.3)	830 (0.4)	111 (0.4)	719 (0.4)
Cardiac Arrhythmia	6479 (27.2)	5940 (28.3)	539 (18.7)	1714 (12.7)	1216 (14.1)	498 (10.2)	459 (15.7)	175 (20.0)	284 (13.9)	7919 (3.5)	2497 (9.8)	5422 (2.7)
Congestive Heart Failure	1211 (5.1)	1121 (5.3)	90 (3.1)	513 (3.8)	379 (4.4)	134 (2.7)	85 (2.9)	37 (4.2)	48 (2.4)	1890 (0.8)	774 (3.0)	1116 (0.6)
Depression	2690 (11.3)	2347 (11.2)	343 (11.9)	1076 (8.0)	595 (6.9)	481 (9.8)	257 (8.8)	66 (7.8)	191 (9.4)	21 517 (9.6)	2495 (9.8)	19 022 (9.5)
Hypothyroidism	2755 (11.6)	2441 (11.6)	314 (10.9)	1345 (10.0)	744 (8.6)	601 (12.3)	260 (8.9)	75 (8.6)	185 (9.1)	21 891 (9.7)	3993 (15.7)	17 898 (9.0)
AIDS/HIV	25 (0.1)	17 (0.08)	8 (0.3)	6 (0.04)	4 (0.05)	2 (0.04)	14 (0.5)	10 (1.1)	4 (0.2)	95 (0.04)	4 (0.02)	91 (0.05)
Liver Disease	501 (2.1)	443 (2.1)	58 (2.0)	350 (2.6)	258 (3.0)	92 (1.9)	990 (34.0)	500 (57.2)	490 (24.0)	1711 (0.8)	447 (1.8)	1264 (0.6)
Pulmonary Circulation Disorder	485 (2.0)	434 (2.1)	51 (1.8)	154 (1.1)	113 (1.3)	41 (0.8)	43 (1.5)	20 (2.3)	23 (1.1)	786 (0.4)	363 (1.4)	423 (0.2)
Peripheral Vascular Disorder	2072 (8.7)	1989 (9.5)	83 (2.9)	397 (2.9)	288 (3.3)	109 (2.2)	67 (2.3)	31 (3.6)	36 (1.8)	1074 (0.5)	371 (1.5)	703 (0.4)
Rheumatoid Arthritis	812 (3.4)	721 (3.4)	91 (3.2)	238 (1.8)	102 (1.2)	136 (2.8)	52 (1.8)	14 (1.6)	38 (1.9)	3361 (1.5)	492 (1.9)	2869 (1.4)
Renal Failure	1436 (6.0)	1325 (6.3)	111 (3.9)	537 (4.0)	367 (4.3)	170 (3.5)	126 (4.3)	60 (6.9)	66 (3.2)	2039 (0.9)	816 (3.2)	1223 (0.6)
Valvular Disease	1144 (4.8)	1039 (5.0)	105 (3.6)	483 (3.6)	309 (3.6)	174 (3.6)	113 (3.9)	43 (4.9)	70 (3.4)	4210 (1.9)	805 (3.2)	3405 (1.7)
Cerebrovascular Disease	748 (3.1)	705 (3.4)	43 (1.5)	204 (1.5)	155 (1.8)	49 (1.0)	34 (1.2)	16 (1.8)	18 (0.9)	742 (0.3)	237 (0.9)	505 (0.3)
Myocardial Infarction	1830 (7.7)	1711 (8.2)	119 (4.1)	580 (4.3)	433 (5.0)	147 (3.0)	105 (3.6)	42 (4.8)	63 (3.1)	1705 (0.8)	554 (2.2)	1151 (0.6)

**Table 3:** Hospital Characteristics of the Study Groups with Surgical Resections

	Lung Lobectomy			LAR			Liver Wedge Resection			Total Hysterectomy		
	Overall *N* = 23 858 N (%)	Cancer *N* = 20 976 N (%)	No Cancer *N* = 2882 N (%)	Overall *N* = 13 522 N (%)	Cancer *N* = 8627 N (%)	No Cancer *N* = 4895 N (%)	Overall *N* = 2916 N (%)	Cancer *N* = 874 N (%)	No Cancer *N* = 2042 N (%)	Overall *N* = 225 075 N (%)	Cancer *N* = 25 511 N (%)	No Cancer *N* = 199 564 N (%)
US Geographic Region												
Midwest	5103 (21.4)	4528 (21.6)	575 (20.0)	2353 (17.4)	1588 (18.4)	765 (15.6)	346 (11.9)	82 (9.4)	264 (12.9)	50 856 (22.6)	5690 (22.3)	45 166 (22.6)
Northeast	4274 (17.9)	3723 (17.8)	551 (19.1)	2803 (20.7)	1781 (20.6)	1022 (20.9)	1096 (37.6)	429 (49.1)	667 (32.7)	31 834 (14.1)	6392 (25.1)	25 442 (12.8)
South	10 223 (42.9)	8952 (42.7)	1271 (44.1)	5770 (42.7)	3536 (41.0)	2234 (45.6)	988 (33.9)	224 (25.6)	764 (37.4)	94 801 (42.1)	8631 (33.8)	86 170 (43.2)
West	4258 (17.9)	3773 (18.0)	485 (16.8)	2596 (19.2)	1722 (20.0)	874 (17.9)	486 (16.7)	139 (15.9)	347 (17.0)	47 584 (21.1)	4798 (18.8)	42 786 (21.4)
Teaching Status												
No	11 113 (46.6)	9902 (47.2)	1211 (42.0)	7739 (57.2)	4729 (54.8)	3010 (61.5)	540 (18.5)	108 (12.4)	432 (21.1)	135 942 (60.4)	9497 (37.2)	126 445 (63.4)
Yes	12 745 (53.4)	11 074 (52.8)	1671 (58.0)	5783 (42.8)	3898 (45.2)	1885 (38.5)	2376 (81.5)	766 (87.6)	1610 (78.8)	89 133 (39.6)	16 014 (62.8)	73 119 (36.6)
Rural/Urban												
Rural	1632 (6.8)	1477 (7.0)	155 (5.4)	1009 (7.5)	766 (8.9)	243 (5.0)	53 (1.8)	14 (1.6)	39 (1.9)	21 687 (9.6)	785 (3.1)	20 902 (10.5)
Urban	22 226 (93.2)	19 499 (93.0)	2727 (94.6)	12 513 (92.5)	7861 (91.1)	4652 (95.0)	2863 (98.2)	860 (98.4)	2003 (98.1)	203 388 (90.4)	24 726 (96.9)	178 662 (89.5)
Bed Size												
Small	757 (3.2)	687 (3.3)	70 (2.4)	517 (3.8)	345 (4.0)	172 (3.5)	40 (1.4)	6 (0.7)	34 (1.7)	13 101 (5.8)	546 (2.1)	12 555 (6.3)
Medium	2394 (10.0)	2122 (10.1)	272 (9.4)	1727 (12.8)	1174 (13.6)	553 (11.3)	178 (6.1)	55 (6.3)	123 (6.0)	37 473 (16.7)	3776 (14.8)	33 697 (16.9)
Large	20 707 (86.8	18 167 (86.6)	2540 (88.1)	11 278 (83.4)	7108 (82.4)	4170 (85.2)	2698 (92.5)	813 (93.0)	1885 (92.3)	174 501 (77.5)	21 189 (83.1)	153 312 (76.8)
Surgical Volume												
1–100	2656 (11.1)	2377 (11.3)	279 (9.7)	239 (1.8)	172 (2.0)	67 (1.4)	1257 (43.1)	291 (33.3)	966 (47.3)	4273 (1.9)	185 (0.7)	4088 (2.1)
101–300	8080 (33.9)	7203 (34.3)	877 (30.4)	1407 (10.4)	986 (11.4)	421 (8.6)	672 (23.1)	193 (22.1)	479 (23.5)	17 775 (7.9)	1102 (4.3)	16 673 (8.4)
300–500	5654 (23.7)	4998 (23.8)	656 (22.8)	1392 (10.3)	971 (11.3)	421 (8.6)	460 (15.8)	120 (13.7)	340 (16.7)	22 789 (10.1)	1240 (4.9)	21 549 (10.8)
≥500	7468 (31.3)	6398 (30.5)	1070 (37.1)	10 484 (77.5)	6498 (75.3)	3986 (81.4)	527 (18.1)	270 (30.9)	257 (12.6)	180 238 (80.1)	22 984 (90.1)	157 254 (78.8)
Type of Cost Source												
Procedural Records	18 101 (75.9)	15 903 (75.8)	2198 (76.3)	9722 (71.9)	6087 (70.6)	3635 (74.3)	2343 (80.4)	705 (80.7)	1638 (80.2)	162 325 (72.1)	19 374 (75.9)	142 951 (71.6)
Cost to Charge Ratio	5757 (24.1)	5073 (24.2)	684 (23.7)	3800 (28.1)	2540 (29.4)	1260 (25.7)	573 (19.7)	169 (19.3)	404 (19.8)	62 750 (27.9)	6137 (24.1)	56 613 (28.4)
Surgical Approach												
Open	14 161 (59.4)	12 593 (60.0)	1568 (54.4)	-	-	-	-	-	-	118 255 (52.5)	17 305 (67.8)	100 950 (50.6)
Minimally Invasive^a^	9697 (40.6)	8383 (40.0)	1314 (45.6)	-	-	-	-	-	-	106 820 (47.5)	8206 (32.2)	98 614 (49.4)

^a^Minimally Invasive approach includes: Lung lobectomy [Video-assisted thoracoscopic surgery (VATS)]; Hysterectomy [laparoscopic, laparoscopically assisted vaginal hysterectomy (LAVH), vaginal)]

### Hospital resource utilization, associated costs, and complications

Unadjusted hospital resource utilization, associated costs, and complications for the overall populations and the stratification by year of hospitalization are shown in Tables 4, 5, 6, 7.

**Table 4:** Hospital Resource Utilization, Costs, and Complications of Patients with Lung Lobectomy

	Overall	2008	2009	2010	2011	2012	2013	2014
Sample Size (N)	23 858	3320	3494	3110	3341	3579	3557	3457
Operating Room Time (minutes)								
Mean (SD)	242.4 (165.1)	241.4 (111.5)	239.8 (105.3)	234.0 (97.6)	233.5 (91.7)	257.7 (314.3)	245.9 (187.3)	242.4 (90.3)
Hospital Length of Stay (days)								
Mean (SD)	7.2 (6.0)	7.7 (6.1)	7.5 (5.8)	7.3 (6.0)	7.3 (6.2)	7.1 (7.0)	6.9 (5.4)	6.8 (5.5)
Hospital Costs								
Mean (SD)	$26 661 ($79 739)	$26 690 ($22 340)	$26 559 ($21 411)	$25 974 ($23 677)	$27 049 ($21 703)	$29 252 ($199 562)	$25 911 ($18 774)	$25 065 ($19 357)
Complications^a^								
Bleeding (%)	9.8	7.0	7.9	8.9	9.1	11.0	12.5	11.5
Infections (%)	8.5	9.8	8.7	9.0	8.0	7.9	8.3	7.7
Cancer status								
Yes (%)	87.9	89.6	88.0	86.8	87.7	87.5	87.5	88.4

^a^During hospitalizations. *SD* Standard deviation

**Table 5:** Hospital Resource Utilization, Costs, and Complications of Patients with LAR

	Overall	2008	2009	2010	2011	2012	2013	2014
Sample Size (N)	13 522	1921	1761	1608	1619	1927	2411	2275
Operating Room Time (minutes)								
Mean (SD)	235.6 (217.7)	223.8 (88.0)	220.1 (92.7)	222.3 (94.2)	217.4 (90.3)	259.7 (523.0)	242.5 (103.4)	251.3 (102.8)
Hospital Length of Stay (days)								
Mean (SD)	6.3 (4.7)	6.7 (4.5)	6.6 (5.2)	6.6 (5.2)	6.2 (4.2)	6.0 (4.2)	6.2 (4.8)	6.1 (4.6)
Hospital Costs								
Mean (SD)	$18 947 ($15 572)	$18 774 ($13 010)	$19 234 ($19 266)	$18 664 ($14 548)	$18 441 ($13 995)	$18 713 ($15 857)	$19 464 ($16 334)	$19 083 ($15 043)
Complications^a^								
Bleeding (%)	9.0	7.0	8.3	8.9	9.5	8.6	10.3	10.0
Infections (%)	7.2	8.3	7.3	7.3	7.8	6.5	7.3	6.2
Cancer status								
Yes (%)	63.8	64.9	64.7	61.5	65.3	61.2	63.7	65.1

^a^During hospitalizations. *LAR* Lower anterior resection, *SD* Standard deviation

**Table 6:** Hospital Resource Utilization, Costs, and Complications of Patients with Liver Wedge Resection

	Overall	2008	2009	2010	2011	2012	2013	2014
Sample Size (N)	2916	489	481	350	331	418	403	444
Operating Room Time (minutes)								
Mean (SD)	280.5 (132.4)	298.3 (150.0)	282.0 (131.4)	278.6 (112.1)	294.6 (145.2)	277.7 (142.4)	264.3 (112.5)	266.7 (120.9)
Hospital Length of Stay (days)								
Mean (SD)	6.4 (6.1)	7.0 (6.6)	6.6 (6.5)	6.6 (6.1)	6.6 (6.2)	6.4 (6.4)	6.1 (6.1)	5.7 (4.7)
Hospital Costs								
Mean (SD)	$25 738 ($27 712)	$27 062 ($26 558)	$25 688 ($24 673)	$25 011 ($22 077)	$30 169 ($37 362)	$26 657 ($33 936)	$23 137 ($20 502)	$23 098 ($26 391)
Complications^a^								
Bleeding (%)	11.6	7.0	10.2	10.0	17.2	13.4	12.2	13.3
Infections (%)	8.7	9.8	9.4	10.0	9.7	8.1	6.2	7.7
Cancer status								
Yes (%)	30.0	30.9	34.5	19.4	22.4	28.5	31.5	38.1

^a^During hospitalizations. *LAR* Lower anterior resection, *SD* Standard deviation

**Table 7:** Hospital Resource Utilization, Costs, and Complications of Patients with Total Hysterectomy

	Overall	2008	2009	2010	2011	2012	2013	2014
Sample Size (N)	225 075	43 462	42 778	32 383	31 240	30 423	24 139	20 650
Operating Room Time (minutes)								
Mean (SD)	162.7 (169.7)	148.6 (68.4)	151.5 (71.9)	153.5 (71.6)	163.8 (151.7)	193.3 (359.7)	169.1 (86.7)	175.3 (220.4)
Hospital Length of Stay (days)								
Mean (SD)	2.2 (1.8)	2.3 (1.8)	2.2 (1.9)	2.1 (1.6)	2.1 (1.8)	2.2 (1.8)	2.2 (2.0)	2.3 (1.9)
Hospital Costs								
Mean (SD)	$8894 ($7003)	$8373 ($5192)	$8518 ($6068)	$8565 ($5482)	$9176 ($6670)	$9410 ($7779)	$9532 ($10 690)	$9355 ($7882)
Complications^a^								
Bleeding (%)	5.1	4.5	4.6	4.4	4.9	5.3	6.3	7.0
Infections (%)	1.2	1.4	1.3	1.1	1.1	1.1	1.2	1.2
Cancer status								
Yes (%)	11.3	10.2	10.5	10.0	11.4	12.5	13.1	13.7

^a^During hospitalizations. *LAR* Lower anterior resection, *SD* Standard deviation

#### Patients undergoing lung lobectomy

Among the overall study population that underwent lung lobectomy, mean ORT was 242.4 min, mean LOS was 7.2 days and the mean total hospital cost was $26 661. Mean hospital LOS showed a steady decrease over time, decreasing from 7.7 days in 2008 to 6.8 days in 2014. Mean ORT and hospital costs did not show a particular trend; they both peaked in 2012 (257.7 min, $29 252) before decreasing to an average ORT of 242.4 min and costs of $25 065 in 2014. Among patients who underwent lung lobectomy, bleeding and infection rates were 9.8%, and 8.5% respectively. The proportions of patients with bleeding (7.0% to 11.5%) numerically increased from the years 2008 to 2014, while the proportions of those with infections numerically decreased (9.8% to 7.7%).

#### Patients undergoing LAR

Among the overall study population that underwent LAR, mean ORT was 235.6 min, mean LOS was 6.3 days and the mean total hospital cost was $18 947. Mean hospital LOS showed a gradual decrease from 6.7 days in 2008 to 6.1 days in 2014. Mean hospital costs stayed relatively stable between the years 2008 and 2014; however, ORT varied with a steady increase in the earlier years (from 223.8 min in 2008 to 259.7 min in 2012). Among patients who underwent LAR, bleeding occurred in 9.0% and infection in 7.2% during their hospitalization. The proportion of patients with bleeding numerically increased from 7.0% in 2008 to 10.0% in 2014. The occurrence of infection numerically decreased from 8.3% in 2008 to 6.2% in 2014.

#### Patients undergoing liver wedge resection

Among the overall study population that underwent liver wedge resection, the mean ORT was 280.5 min and mean LOS was 6.4 days. Total mean hospital cost was $25 738. All 3 resource utilization parameters showed a decrease from 2008 (mean LOS = 7.0 days; mean ORT = 298.3 min; mean cost = $27 062) to 2014 (mean LOS = 5.7 days; mean ORT = 266.7 min; mean cost = $23 098); other than an odd spike for costs in 2011 ($30 169). Among patients who underwent liver wedge resection, bleeding occurred in 11.6% and infection in 8.7% during their hospitalization for resection. The proportion of patients with bleeding numerically increased from 7.0% in 2008 to 13.3% in 2014. The occurrence of infections numerically decreased from 9.8% in 2008 to 7.7% in 2014.

#### Patients undergoing total hysterectomy

Among the overall study population that underwent total hysterectomy, mean ORT was 162.7 min and mean LOS was 2.2 days. Total mean hospital cost was $8894. Both mean ORT and costs increased from 2008 (148.6 min; $8373) to 2014 (175.3 min; $9355). Mean hospital LOS remained relatively constant between the years 2008 and 2014. Complication rates were relatively low among patients who underwent total hysterectomy; bleeding occurred in 5.1% and infection in 1.2% during hospitalization. Similar to the trend in other resections, the proportion of patients with bleeding numerically increased from 4.5% in 2008 to 7.0% in 2014. The occurrence of infection remained relatively constant between the years 2008 and 2014.

### Incremental effect of cancer on hospital ORT, LOS, costs, and complications after adjusting for patient, procedural, and hospital characteristics

**Fig. 1:**
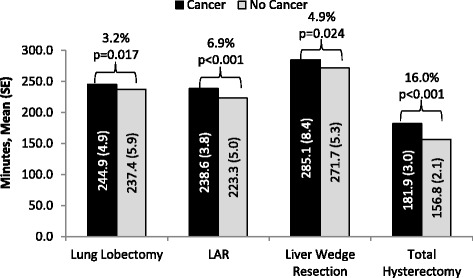
Adjusted Mean Operating Room Times of the Study Groups with Surgical Resections. Percentage indicates incremental impact of cancer. SE: Standard error; LAR: Lower anterior resection

**Fig. 2:**
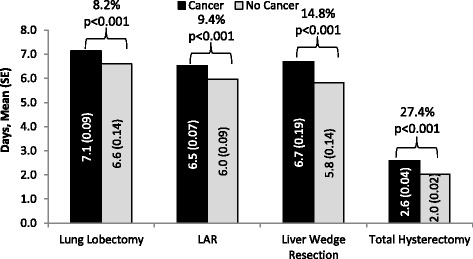
Adjusted Mean Length of Hospital Stays of the Study Groups with Surgical Resections. Percentage indicates incremental impact of cancer. SE: Standard error; LAR: Lower anterior resection

**Fig. 3:**
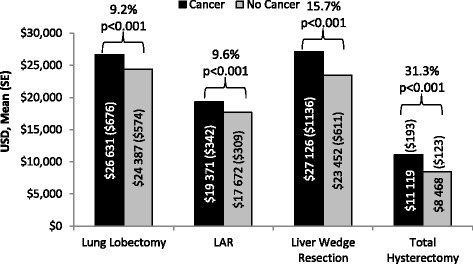
Adjusted Mean Hospital Costs of the Study Groups with Surgical Resections. Percentage indicates incremental impact of cancer. SE: Standard error; LAR: Lower anterior resection

The incremental effect of cancer on hospital resource utilization in the four surgical resections are shown in Figs. 1, 2, 3 and the incremental effect of cancer on complications in shown in Table 8.

**Table 8:** Incremental Effect of Cancer on Risks for Complications During Hospitalization

	Lung Lobectomy	LAR	Liver Wedge Resection	Total Hysterectomy
Bleeding				
Odds Ratio	1.24	1.14	1.17	1.57
Confidence Interval	1.08–1.43	0.98–1.34	0.87–1.58	1.41–1.74
Infection				
Odds Ratio	1.48	1.01	1.76	1.76
Confidence Interval	1.21–1.80	0.87–1.19	1.27–2.43	1.54–2.01

*LAR* Lower anterior resection

#### Patients undergoing lung lobectomy

Among patients who underwent lung lobectomy, the mean ORT (244.9 vs. 237.4 min, *p* = 0.017), mean LOS (7.1 vs. 6.6 days, *p* < 0.001), and mean hospital cost ($26 631 vs. $24 387, *p* < 0.001) were statistically higher by 3.2%, 8.2%, and 9.2%, respectively for patients with cancer in comparison to patients without cancer. The risks for bleeding (OR: 1.24; 95% CI: 1.08–1.43), and infection (OR: 1.48; 95% CI: 1.21–1.80) among this study group were significantly greater for patients with cancer relative to those without cancer.

#### Patients undergoing LAR

Among patients who underwent LAR, the mean ORT (238.6 vs. 223.3 min, *p* < 0.001), mean LOS (6.5 vs. 6.0 days, *p* < 0.001), and mean hospital cost ($19 371 vs. $17 672, *p* < 0.001) were statistically higher by 6.9%, 9.4%, and 9.6%, respectively for patients with cancer in comparison to patients without cancer. The risks for bleeding and infection among this study group were not significantly different for patients with cancer relative to those without cancer.

#### Patients undergoing liver wedge resection

Among patients who underwent liver wedge resection, the mean ORT (285.1 vs. 271.7 min, *p* = 0.02), mean LOS (6.7 vs. 5.8 days, *p* < 0.001), and mean hospital cost ($27 126 vs. $23 452, *p* < 0.001) were statistically higher by 4.9%, 14.8%, and 15.7%, respectively for patients with cancer in comparison to patients without cancer. The risk for infection (OR: 1.76; 95% CI: 1.27–2.43, *p* < 0.001) among this study group was greater for patients with cancer relative to those without cancer, although the risk for bleeding was not statistically significantly different (OR: 1.17; 95% CI: 0.87–1.58).

#### Patients with total hysterectomy

Among patients who underwent total hysterectomy, the mean ORT (181.9 vs. 156.8 min, *p* < 0.001), mean LOS (2.6 vs. 2.0 days, *p* < 0.001), and mean hospital cost ($11 119 vs. $8468, *p* < 0.001) were statistically higher by 16.0%, 27.4%, and 31.3%, respectively for patients with cancer in comparison to patients without cancer. The risks for bleeding (OR: 1.57; 95% CI: 1.41–1.74) and infection (OR: 1.76; 95% CI: 1.54–2.01) among this study group were significantly greater for patients with cancer relative to those without cancer.

### Sensitivity analyses of patients with different surgical approaches and impact of cancer on outcomes

The results of the sensitivity analyses are shown in Table 9. Among patients who underwent lung lobectomy with an open approach (*N* = 14 161) mean LOS and hospital cost were statistically higher by 6.2%, and 5.4%, respectively for patients with cancer in comparison to patients without cancer. The risk for infection was significantly greater for patients with cancer vs. patients without cancer. These results are generally consistent with the results found in the overall population undergoing lung lobectomy; however, the magnitude of incremental economic burden associated with cancer was lower compared to the overall sample.

**Table 9:** Sensitivity Analyses: Incremental Effect of Cancer on Hospital Resource Utilization, Costs, and Risks for Complications During Hospitalization

	Lung Lobectomy					
	Open (*N* = 14 161)			VATS (*N* = 9697)		
	Cancer	No Cancer	*p*-value	Cancer	No Cancer	*p*-value
Operating Room Time						
(Mean, minutes)	248.3	248.6	0.961	254.4	236.7	<0.001
2003Hospital Length of Stay						
(Mean, days)	8.1	7.6	0.002	5.8	5.3	<0.001
Hospital Costs						
(Mean)	28 028	26 593	0.006	25 690	23 091	<0.001
Complications: Cancer vs. No Cancer						
Bleeding						
Odds Ratio	1.14			1.55		
95% Confidence Interval	0.96–1.36			1.17–2.04		
Infection						
Odds Ratio	1.41			1.63		
95% Confidence Interval	1.11–1.80			1.20–2.22		
	Total Hysterectomy					
	Open (*N* = 118 255)			Other^a^ (*N* = 106 820)		
	Cancer	No Cancer	*p*-value	Cancer	No Cancer	*p*-value
Operating Room Time						
(Mean, minutes)	178.5	152.3	<0.001	192.1	165.5	<0.001
Hospital Length of Stay						
(Mean, days)	3.6	2.7	<0.001	1.6	1.6	0.032
Hospital Costs						
(Mean)	11 396	8485	<0.001	10 424	8606	<0.001
Complications: Cancer vs. No Cancer						
Bleeding						
Odds Ratio	1.65			1.13		
95% Confidence Interval	1.48–1.84			0.93–1.37		
Infection						
Odds Ratio	1.96			0.91		
95% Confidence Interval	1.70–2.27			0.66–1.24		

^a^Laparoscopic, laparoscopically assisted vaginal hysterectomy, vaginal

*VATS* Video-assisted thoracoscopic surgery

Among patients who underwent lung lobectomy with VATS (*N* = 9697) the mean ORT, LOS, and hospital cost were statistically higher by 7.4%, 10.9%, and 11.3%, respectively for patients with cancer in comparison to patients without cancer. The risks for bleeding, and infection were significantly greater for patients with cancer vs. patients without cancer. These results are generally consistent with the results found in the overall population undergoing lung lobectomy; however, the incremental burden of cancer was higher in the sub-group of patients undergoing VATS.

Among patients who underwent total hysterectomy with an open approach (*N* = 118 255) the mean ORT, LOS, and hospital cost were statistically higher by 17.2%, 34.3%, and 34.3%, respectively for patients with cancer in comparison to patients without cancer. The risks for bleeding and infection were significantly greater for patients with cancer vs. patients without. These results are generally consistent with the results found in the overall population undergoing hysterectomy; however, the incremental burden associated with cancer was higher in patients undergoing an open approach.

Among patients who underwent total hysterectomy by a minimally invasive approach (*N* = 106 820; i.e., laparoscopic, vaginal, LAVH) the mean ORT, LOS, and hospital cost were statistically higher by 16.1%, 4.6%, and 21.1%, respectively for patients with cancer in comparison to patients without cancer. These results are generally consistent with the results found in the overall population undergoing hysterectomy; however, the association of cancer and outcomes was lower than in the overall sample. In contrast to results of the overall population of women who underwent total hysterectomy, the risks for bleeding and infection were similar for patients with cancer and those without cancer.

## Discussion

Based on this large hospital database analysis, patients who undergo lung lobectomy, LAR, liver wedge resections, or total hysterectomy for a cancer indication were found to have significantly increased hospital resource utilization compared to these same surgeries for benign indications. This study was unique in terms of being one of the few large scale analyses in the real-world setting of a large sample of patients undergoing surgeries of diverse anatomies (lung, rectum, liver and uterus) over multiple years. The results of the sensitivity analyses of the subpopulations of patients with lung lobectomy and total hysterectomy stratified by surgical approach (traditional open vs minimally invasive) were generally consistent with that of the overall corresponding study groups. The impact of having a cancer indication on outcomes was greater among patients who underwent VATS lobectomy compared to an open surgical approach. Contrastingly, the impact of cancer on outcomes was less among women who underwent minimally invasive total hysterectomy compared to an open surgical approach. Across all surgical resections, patients with a cancer indication were older and more likely to have comorbid conditions. These differences in perioperative risk factors may potentially contribute to greater hospital resource use and higher complication rates of cancer patients undergoing surgical resections [9]. However, after adjusting for differences in patient characteristics, a cancer indication was independently associated with greater hospital resource use (LOS and ORT), higher total hospital costs, and greater bleeding and infection complication rates in this study. These results quantify the importance of taking into consideration critical patient characteristics such as the indication for surgery when selecting the most appropriate clinical and surgical strategies.

For the majority of surgical resections evaluated in this study, recent comparative data on hospital resource utilization and costs are limited, especially in the context of patients with cancer. A study of 3818 patients with lung cancer who underwent VATS lobectomy between 2009 and 2011 reported an average ORT of 252 min, LOS of 5.8 days, and hospital cost of $20 477 [10]. For comparison, in our study patients with VATS lobectomy for cancer had an average ORT of 254.4 min, LOS of 5.8 days, and hospital cost of $25 690. The greater hospital costs observed in our study is likely explained by the different study years included and that our costs were inflation adjusted to 2014 USD. Otherwise, the results are quite consistent with those of Swanson et al. [10]. A nationwide study of patients with colon cancer between 2008 and 2009 reported an average LOS of 5.5 days and an average hospital cost of $15 807 for patients who underwent laparoscopic surgery [11]. In our study, patients with specific LAR of the rectum had an average LOS of 6.5 days, and an average hospital cost of $19 371. More recent estimates or data specific to LAR of the rectum are not available in the literature. In regard to hysterectomy, our results are similar to those of Wallace et al., who found that of 59 525 women who underwent hysterectomies, complications and ORTs were more prevalent among those with gynecologic malignancies in comparison to women with benign conditions [8]. Furthermore, there is evidence that women with gynecologic cancers more frequently undergo bilateral salpingoophorectomy than women with benign disease [12].

The greater hospital resource use, costs, and complication rates associated with oncologic surgical resections relative to benign indications could be attributed to several differences in the surgical techniques required, extended tissue resection for adequate margins, lymph node dissection for staging, and/or the resection of ancillary tissues [13–15]. Other factors unique to cancer patients include the potential for prior therapies related to their cancer care, such as surgical procedures or neo-adjuvant chemo-radiation which can affect the tissue quality or cause adhesions making the dissection more challenging and may contribute to lengthier ORTs and greater hospital resource use [13].

This large scale analysis in the real-world setting provides information for clinicians, hospital administrators, and other healthcare stakeholders about the economic burdens of the evaluated surgical resections. Variation in outcomes based on cancer as opposed to other indications may have major implications on assessing hospital performance and it is critical to account for indication specific data when evaluating hospital quality and benchmarking hospital performance [7]. The results of our study imply that when hospitals are risk stratifying surgical procedures, cancer is an important marker of complexity. These challenging patients and surgically complex procedures require innovative improvements in clinical care and may potentially benefit from improved medical devices and surgical techniques to further optimize their surgical outcomes.

Our data also provide valuable information on the trends of surgical outcomes among a large population of patients with surgical resections over a period of 7 years. In the study groups of patients with lung lobectomy, LAR, and liver wedge resection, the length of hospital stay declined from 2008 to 2014; this could be attributed to secular improvements in surgical technique and the increased utilization of minimally invasive techniques in conjunction with the proliferation of prospective payment systems that incentivize hospitals to reduce LOS. In regard to complication rates, the incidence of bleeding during hospitalization increased from years 2008 to 2014 across all study groups with organ resections, while the incidence of infection decreased among those with lung, colon, and liver resections. The decrease in infection rates could potentially be attributed to improved hospital practices. An increase in the incidence of bleeding is significant, especially for patients with lung lobectomy, as bleeding has been associated with increased hospital length of stay and a substantial increase in hospital costs among patients with lung resection surgery in the US [16].

### Limitations

This study was a retrospective study and the results are observational. Measurements of patient and hospital characteristics were evaluated descriptively and not statistically compared. Patient data in the study was only representative of hospital costs and excluded outpatient healthcare utilization and costs. Also, this study reflects surgeries conducted in the inpatient setting (>24 h involving an overnight stay) and does not capture the growing volume of surgeries conducted in outpatient settings or stand-alone ambulatory surgical centers. Clinically important missing variables which may represent unmeasured confounders, such as stage of cancer, actual anatomic location, prior operations, prior radiation/chemotherapy, missing approach for liver wedge resection and LAR, measures of patient comorbidity burden, such as American Society of Anesthesiologist (ASA) score or nutritional status, and devices/technology utilized were not evaluated. Although the study controlled for multiple factors including comorbidity indices and conditions; total risk adjustment may not be feasible in such studies. Also, the study focused only on general complications that occur in most surgeries (bleeding and infection) and did not examine important anatomically specific complications such as air leaks for thoracic lobectomies and anastomotic leaks for LAR.

## Conclusion

In this analysis, we found that patients who underwent lung lobectomy, lower anterior resection of the rectum (LAR), liver wedge resection or total hysterectomy for a cancer indication have significantly increased hospital resource utilization compared to these same surgeries for benign indications. Utilizing the most effective clinical strategies inclusive of innovative surgical technologies and techniques may help reduce the economic burden among complex oncologic surgical resections.
